
JOURNAL INFORMATION
==============================
NLM Title Abbreviation: ScientificWorldJournal
Journal ID: ScientificWorldJournal
NLM Journal ID: 101131163
Journal ID: TSWJ

The Scientific World Journal

ISSN: 2356-6140
EISSN: 1537-744X
Publisher: Wiley

ARTICLE INFORMATION
==============================
PMCID: PMC6083930
PMID: 30147570
DOI: 10.1100/tsw.2001.210
Article ID: 292170
Article version: 1
Subjects: Short Report

Expression of Nuclear Orphan Receptors Ngfi-B/Tr3, Nurr1 And Nor-1 after Global Brain Ischemia in Rat

Schmidt-Kastner Rainald 1 2 *
Zetterstr m Rolf 3
Hakim Antoine M. 1
1 Neuroscience Research Institute, University of Ottawa, Ottawa, Canada
2 Department of Neurology (D4-5), University of Miami School of Medicine, P.O. Box 016960, Miami, Florida 33101, USA
3 Dept. of Neuroscience, Karolinska Institute, Stockholm, Sweden
*Rainald Schmidt-Kastner: rainald@stroke.med.miami.edu
Publication date: 2001
Electronic publication date: 2001 Jan 12
Volume: 1
First page: 95
Last page: 95
Copyright: Copyright © 2001 Rainald Schmidt-Kastner et al.
Copyright year: 2001
License: This is an open access article distributed under the Creative Commons Attribution License, which permits unrestricted use, distribution, and reproduction in any medium, provided the original work is properly cited.
License URL: https://creativecommons.org/licenses/by/3.0/

Funding: Heart and Stroke Foundation of Canada Deutsche Forschungsgemeinschaft

==============================
Miami *Nature Biotechnology* Short Reports
*TheScientificWorld* (2001) 1 (S3), 95SR
ISSN 1532-2246; DOI 10.1100/tsw.2001.210

### EXPRESSION OF NUCLEAR ORPHAN RECEPTORS NGFI-B/TR3, NURR1 AND NOR-1 AFTER GLOBAL BRAIN ISCHEMIA IN RAT.

Rainald Schmidt-Kastner* (1, 2), Rolf Zetterström (3), and Antoine M. Hakim (1)

Neuroscience Research Institute, University of Ottawa, Ottawa, Canada; (2) Department of Neurology (D4-5), University of Miami School of Medicine, P.O. Box 016960, Miami, Florida 33101, USA; Dept. of Neuroscience, Karolinska Institute, Stockholm, Sweden
* Rainald@stroke.med.miami.edu

**INTRODUCTION**. Neuron-derived orphan receptor (NOR-1) was cloned from cultured embryonal neurons undergoing apoptotic cell death. NOR-1 is related to the nuclear orphan receptors, NGFI-B and NURR1, which interact with the retinoic acid receptor, RXR. NGFI-B and NURR1 are immediate early genes in the ischemic brain, suggesting a role in repair or cell death. Very recently, NGFI-B/TR3 was found to exert a pro-apoptotic effect through mitochondrial targeting and cytochrome release leading to apoptosis (1). Such dual role of NGFI-B (and related orphans) in nuclear and mitochondrial function could be important for neuronal damage in brain ischemia (stroke). We evaluated novel gene expression (mRNA levels) of nuclear orphan receptors after short brain ischemia. This model allows to correlate changes in gene and protein expression with pathological outcome, i.e. selective vulnerability occurs in CA1 neurons of the hippocampus while the adjacent CA3 cells and the dentate granule cells survive.

**METHOD**. Anesthetized rats were subjected to 10 min of four-vessel occlusion and survived for 1, 3, 6, 12 h, and 1, 3 and 7 d (n=3-4 per group); sham controls were prepared in parallel (2). In situ hybridization for NOR-1, NGFI-B and NURR1 mRNAs was carried out using specific antisense oligonucleotides. Emulsion-coated sections were prepared and examined under a low-power dark-field condensor. TUNEL labeling served to visualize DNA fragmentation (2).

**RESULTS**. In control brains, NOR-1, NGFI-B and NURR1 mRNAs were found in CA1 neurons, and NOR-1 and NGFI-B mRNAs in CA3 neurons. Dentate granule cells were entirely negative for all three mRNAs. After ischemia, intense upregulation of NOR-1, NGFI-B and NURR1 mRNAs was seen in the dentate gyrus by 1 h, which then declined by 3 to 6 h. Neurons in CA1 to CA3 showed moderate elevations for NOR-1, NGFI-B and NURR1 mRNAs at 1 to 3 h, but not a later time points. NGFI-B was elevated in cortical cells by 1 to 3 h. Labeling also occurred with a vascular pattern in the cortex indicating expression in endothelial cells. Ischemic cell death occurred in medial CA1 by three days, as indicated by TUNEL labeling; no damage was observed in CA3 or granule cells.

**DISCUSSION**. Nuclear orphan receptors, NOR-1, NGFI-B and NURR1, were rapidly upregulated after short global brain ischemia, indicating a role as immediate early genes. Upregulation occurred in granule cells but much less in CA1 and CA3 regions. Granule cells are resistant to ischemic cell damage, suggesting that the upregulation of the pro-apoptotic

NGFI-B and related nuclear orphan receptors is not detrimental. However, granule cells can show apoptotic changes after withdrawal of adrenal steroids, suggesting that these cells are "at risk". Concomitant upregulation of BDNF and NGF mRNA could provide a protective mechanism for granule cells. Moderate regulation in both vulnerable CA1 and resistant CA3 neurons indicates that novel expression of nuclear orphan receptors does not play a role in ischemic injury. Constitutively expressed NGFI-B, NURR1 and NOR-1 could still participate in the cell death of CA1 neurons, e.g. by a modulation of retinoic acid signaling through interaction of orphan receptors with the retinoic acid receptor RXR.

**ACKNOWLEDGEMENT**. Supported by the Heart Stroke Foundation Ontario (AMH, RS-K) and DFG (Heisenberg-Stipendium, Schm 776/4-2).

**REFERENCES.**

1. Li, H., Kolluri, S.K., Gu, J., Dawson, M.I., Cao, X., Hobbs, P.D., Lin, B., Chen, Q.G., Lu, J.S., Lin, F., Xie, Z., Fontana, J.A., Reed, J.C., and Zhang, X.K. (2000) Science 289, 1159-1164
2. Schmidt-Kastner, R., Fliss, H., and Hakim, A.M. (1997) Stroke 28, 163-170
